# Formation of nuclear bodies by the lncRNA Gomafu-associating proteins Celf3 and SF1

**DOI:** 10.1111/gtc.12169

**Published:** 2014-08-21

**Authors:** Akira Ishizuka, Yuko Hasegawa, Kentaro Ishida, Kaori Yanaka, Shinichi Nakagawa

**Affiliations:** RNA Biology Laboratory, RIKEN2-1 Hirosawa, Wako, Saitama, 351-0198, Japan

## Abstract

Gomafu/MIAT/Rncr2 is a long noncoding RNA that has been proposed to control retinal cell specification, stem cell differentiation and alternative splicing of schizophrenia-related genes. However, how Gomafu controls these biological processes at the molecular level has remained largely unknown. In this study, we identified the RNA-binding protein Celf3 as a novel Gomafu-associating protein. Knockdown of Celf3 led to the down-regulation of Gomafu, and cross-link RNA precipitation analysis confirmed specific binding between Celf3 and Gomafu. In the neuroblastoma cell line Neuro2A, Celf3 formed novel nuclear bodies (named CS bodies) that colocalized with SF1, another Gomafu-binding protein. Gomafu, however, was not enriched in the CS bodies; instead, it formed distinct nuclear bodies in separate regions in the nucleus. These observations suggest that Gomafu indirectly modulates the function of the splicing factors SF1 and Celf3 by sequestering these proteins into separate nuclear bodies.

## Introduction

The genomes of higher vertebrates produce a large number of non-protein-coding transcripts that regulate a variety of cellular processes (reviewed in Kapranov *et al*. 2007; Mercer *et al*. 2009; Rinn & Chang 2012). Parts of these non-protein-coding RNAs are processed into small RNAs that control the expression of target genes through a mechanism known as ‘RNA silencing’. Long noncoding RNAs (lncRNAs) also interact with diverse protein components to form a variety of regulatory machineries. The most well-studied lncRNA, Xist, inactivates one of the two X chromosomes in female mammals by recruiting a chromatin modifying complex to the entire length of the silenced chromosome (reviewed in Brockdorff 2011; Gendrel & Heard 2011). Similarly, certain imprinted genomic loci produce lncRNAs, such as Airn and Kcnq1ot1, that interact with epigenetic regulators and control allele-specific gene expression (reviewed in Mohammad *et al*. 2009). A series of recent studies identified a number of lncRNAs, including HOTAIR, HOTTIP, lncRNAa1-7 and linc-p21, that function as transcriptional activators or repressors in concert with various chromatin modifiers (reviewed in Mercer *et al*. 2009; Rinn & Chang 2012). A genome-wide analysis showed that approximately 20% of human lncRNAs associate with the polycomb complex PRC2 (Khalil *et al*. 2009). These reports suggest that the epigenetic control of gene expression through the modification of chromatin structure is one of the major roles of lncRNAs that are transcribed from the genomes of higher eukaryotes (reviewed in Mercer *et al*. 2009; Rinn & Chang 2012).

Aside from their involvement in the control of epigenetic gene expression, a distinct set of lncRNAs are expressed at extremely high levels and abundantly accumulate in the nucleus, forming specific nuclear bodies (reviewed in Mao *et al*. 2011b; Ip & Nakagawa 2012). meiRNA is one of the first examples of a functional lncRNA-forming nuclear bodies, promoting meiosis in fission yeast (Yamashita *et al*. 1998). Other nuclear body-related lncRNAs include Malat1, which localizes to the nuclear speckles containing various splicing factors (Ji *et al*. 2003; Hutchinson *et al*. 2007; Tripathi *et al*. 2010), Neat1, which functions as an architectural component of nuclear bodies called paraspeckles (Hutchinson *et al*. 2007; Chen & Carmichael 2009; Clemson *et al*. 2009; Sasaki *et al*. 2009; Sunwoo *et al*. 2009), Terra, which localizes to the telomeres and is involved in the maintenance of the chromosomal ends (Feuerhahn *et al*. 2010), and Gomafu (also referred to as MIAT in human or Rncr2 in mouse), which is expressed in specific neuronal cell types and forms novel nuclear bodies that do not overlap with known nuclear compartment markers (Blackshaw *et al*. 2004; Ishii *et al*. 2006; Sone *et al*. 2007). Several independent laboratories have reported that the lncRNA Gomafu has physiological functions in multiple biological processes. For example, specific SNPs in MIAT/Gomafu have been shown to correlate with an increased risk of myocardial infarction, and some of these point mutations in the MIAT transcripts inhibit the association with certain unknown protein factors (Ishii *et al*. 2006). Another study reported that blocking Rncr2/Gomafu function either by shRNA or a novel dominant negative form of Gomafu results in an increase in amacrine and Müller glial cells in the mouse retina, suggesting that Gomafu controls the specification of neural cell types (Rapicavoli *et al*. 2010). In addition, the expression of Gomafu is dynamically regulated during neural stem cell (Mercer *et al*. 2008, 2010) or ES cell differentiation (Sheik Mohamed *et al*. 2010). Gomafu expression is also altered in heroin abusers (Michelhaugh *et al*. 2011). A very recent study suggested that Gomafu is controlled by neuronal activities and is involved in schizophrenia-related alternative splicing (Barry *et al*. 2014). These lines of circumstantial evidence suggest that Gomafu is involved in cellular differentiation as well as the control of neural function. We have recently reported that Gomafu interacts with the splicing factor SF1 through a tandem array of UACUAAC motifs (Tsuiji *et al*. 2011). However, the molecular mechanisms through which Gomafu controls neural function remain largely unknown.

To further characterize the mode of action of Gomafu, we identified additional Gomafu-interacting proteins by screening a custom siRNA library designed against abundant RNA-binding proteins. We suggested that the knockdown of Gomafu-associated proteins might alter the expression or distribution of Gomafu. We observed that knockdown of Celf3 leads to the down-regulation of Gomafu. Immunoprecipitation analysis with antiserum against Celf3 confirmed a specific interaction between Gomafu and Celf3. Interestingly, Celf3 formed novel nuclear bodies (CS bodies) in the neuroblastoma cell line Neuro2A that colocalized with SF1, another Gomafu-interacting protein. However, Gomafu did not accumulate in the CS bodies but was instead separately distributed throughout the nucleus. We propose that Gomafu indirectly modulates the function of RNA-binding proteins in CS bodies by sequestering these proteins in separate regions of the nucleus.

## Results

### lncRNA Gomafu forms a large complex in cells

**Figure 1:**
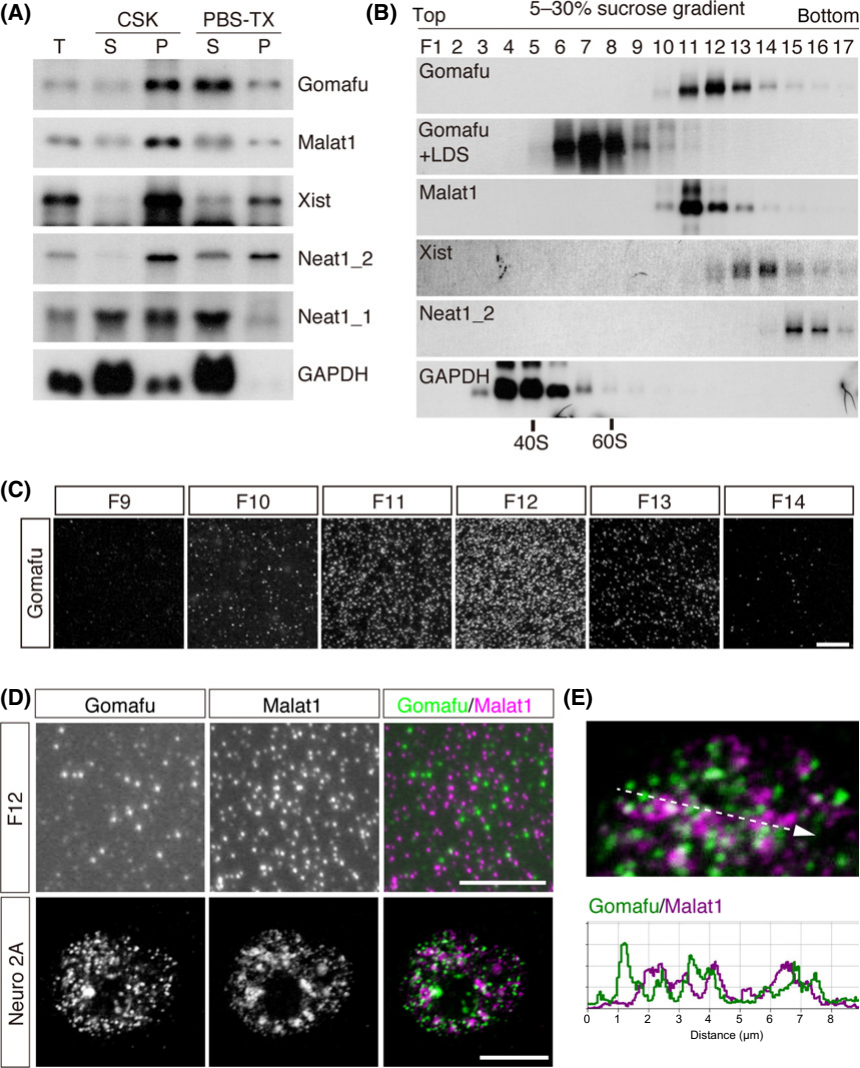
Biochemical analysis of the nuclear lncRNA complex. (A) Northern blot analysis of the solubility of the lncRNAs. Cells were extracted with CSK or PBS-Triton X-100 (PBS-TX), and the RNA prepared from the soluble (S) and insoluble (P) fractions was analyzed by Northern blot. Gomafu and other abundant nuclear lncRNAs (Malat1, Xist and Neat1_1/2) were largely insoluble in the CSK buffer, but a significant amount became soluble in PBS-TX. ‘T’ indicates total RNA recovered from intact cells. (B) Size distribution of the lncRNA complexes. The nuclear lncRNA complexes were sedimented in a 5-30% sucrose gradient in the presence of EDTA, and the RNA prepared from each fraction was analyzed by Northern blot. The positions of the fraction containing the 40S and 60S ribosome are shown at the bottom. It should be noted that the addition of LDS shifted the distribution of Gomafu into the lighter fractions. (C) Fractions 9 to 14 shown in ‘B’ (F9 to F14) were immobilized on PLL-coated glass, and Gomafu was detected by FISH. (D) Simultaneous FISH detection of Gomafu (Green) and Malat1 (Magenta) in F12 and Neuro2A cells by confocal microscopy. (E) A higher magnification image of the Malat1 and Gomafu distribution shown in ‘D’; the intensity profile graph along the line segment shown in the dotted line. Scale bar, 10 μm.

To biochemically characterize the Gomafu complex, we initially determined the buffer conditions that enabled the solubilization of the nuclear matrix-associated components from homogenized nuclei under nondenaturing conditions. As previously reported (Sone *et al*. 2007), PIPES-buffered CSK (cytoskeleton buffer) saline containing a nonionic detergent failed to extract Gomafu RNA, whereas the use of phosphate buffer dramatically increased the RNA solubility (Fig. 1A). The phosphate buffer also solubilized other lncRNA-forming nuclear bodies, including Malat1, Xist and Neat1 (Fig. 1A). We then fractionated the lncRNA complexes on a 5–30% linear sucrose density gradient, and the RNA that was extracted from each fraction was analyzed by Northern blotting to examine the size distribution of the lncRNA complexes. The Gomafu complex sedimented much faster than the 60S ribosome (Fig. 1B), suggesting that this lncRNA formed an extremely large complex. The addition of the ionic detergent LDS dissociated the complex, and Gomafu was fractionated into similar fractions between the 40S and 60S ribosome (Fig. 1B). Other lncRNA complexes also sedimented into the heavy fractions, and Neat1_2, an architectural component of the nuclear bodies known as paraspeckles, was fractionated into the heaviest fraction (Fig. 1B).

We then observed the biochemically fractionated lncRNA complexes microscopically. We immobilized each fraction onto poly-L-lysine (PLL)-coated glass slides and carried out fluorescent *in situ* hybridization (FISH). The intensity of the FISH signals in each fraction correlated with the size distribution of Gomafu as indicated by Northern blot analysis (Fig. 1B, C), suggesting that we could successfully visualize the complex. We then simultaneously detected Malat1 and Gomafu using a fraction that contained both of the lncRNA complexes and found that the FISH signals were independently observed for each lncRNA and were similar to the distribution in the cells (Fig. 1D), suggesting that our biochemical fractionation and immobilization methods maintained the specificity and integrity of the lncRNA complexes (Fig. 1D, E).

### Celf3 associates with Gomafu and regulates Gomafu expression

**Figure 2:**
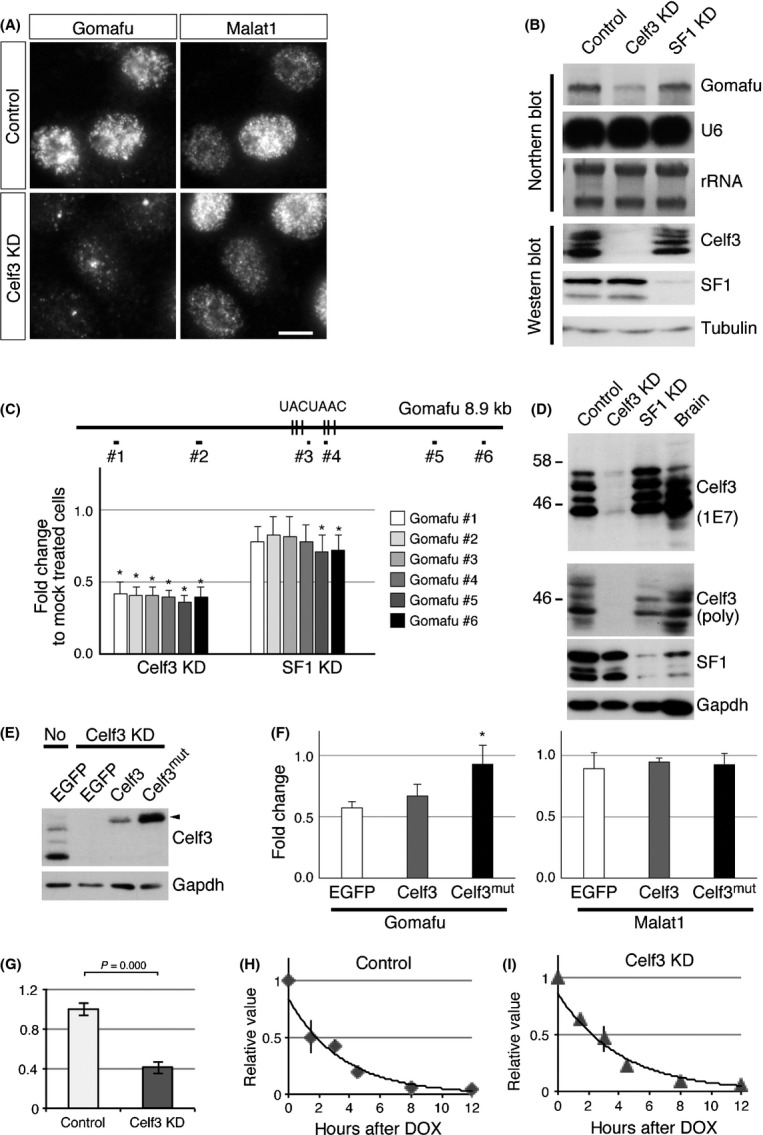
Identification of Celf3 as a Gomafu-binding protein. (A) *In situ* hybridization of Gomafu and Malat1 in cells depleted of Celf3. Knockdown of Celf3 (Celf3 KD) led to a marked reduction in Gomafu levels. (B) Northern blot analysis of Gomafu and U6 in Celf3/SF1 knockdown cells and Western blot analysis confirming the specific knockdown of Celf3 and SF1 in the same samples. rRNA and tubulin signals are shown as loading controls. (C) qPCR analysis of Gomafu in cells treated with siRNAs against Celf3 and SF1. The positions of the primer pairs used for Gomafu quantification are shown at the top (#1 to #6). The vertical bars represent position of the UACUAAC sequence motifs. (D) Western blot analysis of control and Celf3/SF1 knockdown Neuro2A cells or brain lysates using the anti-Celf3 monoclonal antibody 1E7 and polyclonal antibodies against Celf3 (poly), SF1 and Gapdh. (E) Western blot analysis of Celf3 expression in Celf3 KD cells transfected with vectors expressing EGFP, Celf3 and Celf3 containing point mutations resistant to siRNA (Celf3mut). ‘No’ indicates the control cell lysate without siRNA treatment. (F) qPCR analysis of Gomafu and Malat1 in the cells co-transfected with Celf3 siRNA and vectors expressing EGFP, Celf3 and Celf3mut. The reduction of Gomafu upon knockdown of Celf3 is rescued by Celf3mut. (G) qPCR analysis of Gomafu upon knockdown of Celf3 in Neuro2A cells that conditionally express Gomafu under the control of tetracycline-responsive element. (H, I) Measurement of the stability of Gomafu. The expression of Gomafu was measured at the indicated time points after the addition of doxycycline in the control (H) and Celf3-depleted cells (I). Note that the half-life of Gomafu was not greatly affected by Celf3 knockdown. Scale bar, 10 μm. Asterisks indicate *P *<* *0.05 (Student's *t*-test).

The aforementioned biochemical study suggested that Gomafu interacts with multiple proteins to form a large RNP complex. To identify other Gomafu-interacting proteins in addition to SF1 (Tsuiji *et al*. 2011), we screened a custom siRNA library against known abundant RNA-binding proteins (Hasegawa *et al*. 2010). We suggested that the depletion of the candidate proteins might change the subcellular distribution or expression levels of Gomafu. For this screen, we expressed the Gomafu cDNA under the control of the artificial promoter CAG (Niwa *et al*. 1991) in the neuroblastoma cell line Neuro2A because no cultured cell line that endogenously expresses Gomafu is available. Among the 172 RNA-binding proteins, we focused on the RNA-binding protein Celf3 (CUGBP Elav-like family member 3, also referred to as Tnrc4, Brunol1, CAGH4 or ERDA4), which was originally identified as a member of the CUG repeat binding protein 1 family (reviewed in Ladd 2013), because knockdown of this protein led to a marked decrease of exogenous Gomafu as indicated by quantitative PCR (qPCR) (Table S1 in Supporting Information). Decreased levels of Gomafu upon knockdown of Celf3 in Neuro2A cells were confirmed by FISH and Northern blot analysis, whereas the expression of U6 was not affected (Fig. 2A, B). qPCR analyses with multiple primers designed against various regions of Gomafu showed a similar reduction over the entire length of the transcripts, suggesting that Celf3 knockdown did not induce aberrant splicing or 3' processing of Gomafu (Fig. 2C). The reduction of Gomafu expression was rescued by the expression of a mutant Celf3 (Celf3mut) that carried point mutations resistant to the siRNA-mediated knockdown, suggesting that the effect was not caused by off-target effects of the siRNA (Fig. 2E, F).

To study the molecular mechanism by which Celf3 regulates the expression of Gomafu, we examined the stability of Gomafu in cells depleted of Celf3. For this, we established a stable Neuro2A transfectant that conditionally expressed Gomafu under the control of the tetracycline-inducible promoter together with the transactivator Tet-OFF. In this cell line, the expression of Gomafu was shut down upon the addition of doxycycline. Unexpectedly, the half-life of Gomafu in the control cells was approximately 2 h (Fig. 2H), which was much shorter than our previous observations obtained with the transcriptional inhibitor α-amanitin (Sone *et al*. 2007). Because transcriptional inhibition affects various nuclear processes and interferes with the precise measurement of RNA metabolism (Tani *et al*. 2012), the half-life of Gomafu might have been incorrectly predicted in the previous study. We then examined the effect of Celf3 knockdown on the stability of Gomafu. The expression of Gomafu was decreased in this cell line upon Celf3 knockdown (Fig. 2G), suggesting that the decrease in Gomafu expression was independent of the promoter sequences that drive the expression of the exogenous Gomafu. However, the half-life of Gomafu was not affected in the cells depleted of Celf3 (Fig. 2H, I), suggesting that Celf3 regulates the expression of Gomafu at the transcriptional level.

**Figure 3:**
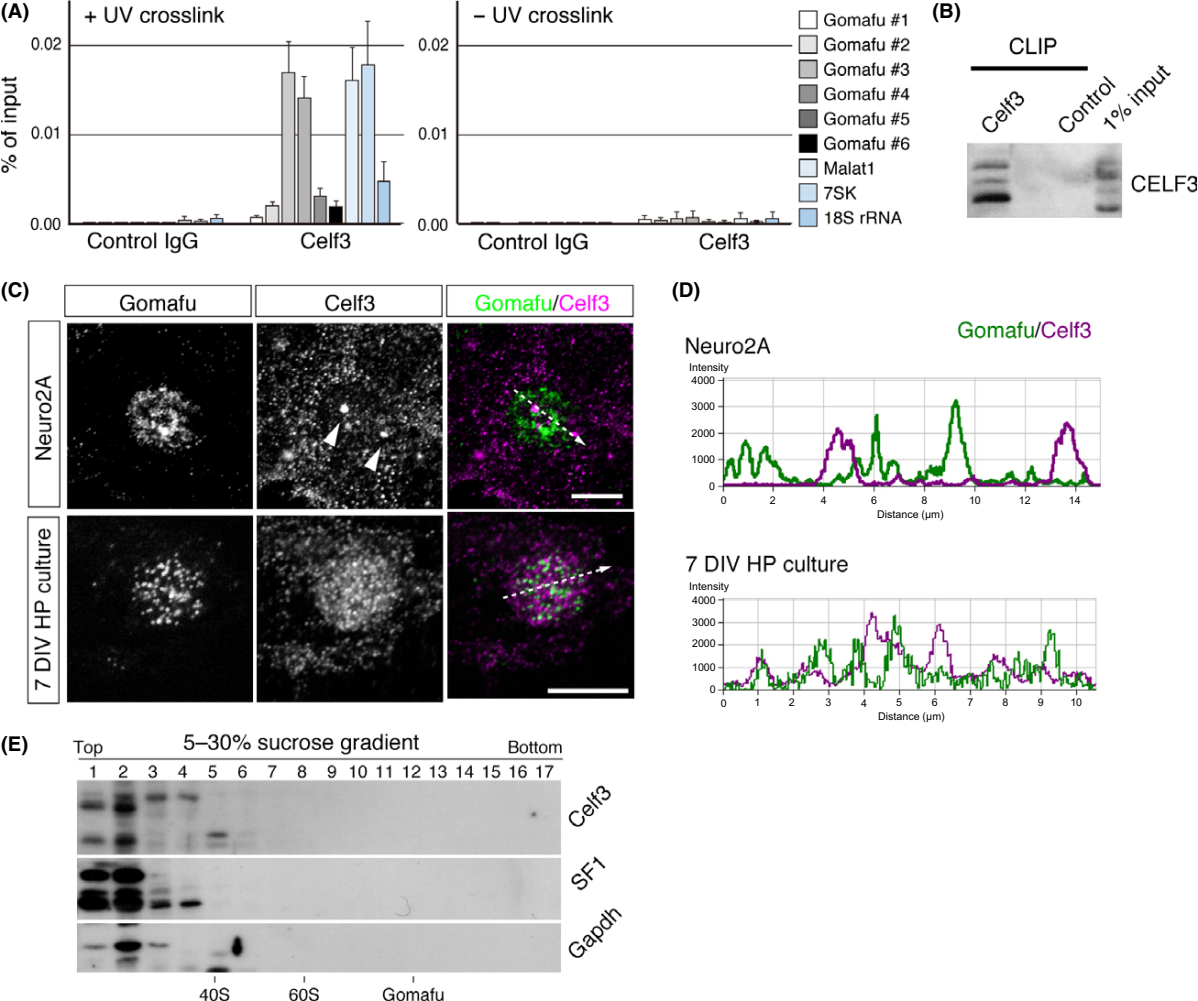
Celf3 directly interacts with Gomafu. (A) CLIP-qPCR analysis of the interactions of Celf3 with Gomafu and other RNAs. Celf3 preferentially associated with the middle region of Gomafu (region #3 and #4 shown in Figure 2C). This interaction disappeared in the absence of UV cross-linking (UV cross-link). Celf3 also interacted with Malat1 and 7SK. (B) Western blot analysis of Celf3 recovered by CLIP. More than 1% of Celf3 was immunoprecipitated. (C) The simultaneous detection of Celf3 (magenta) and Gomafu (green) in Neuro2A cells. (D) Intensity prolife graph of Gomafu and Celf3 signals along the line segment shown in ‘C’. (E) Celf3 and SF1 were not detected in the heavy fractions containing Gomafu separated by sucrose density gradient ultracentrifugation. Arrowheads indicate the site of Celf3 accumulation. Scale bar, 10 μm.

To further investigate the interaction between Celf3 and Gomafu, we immunized mice with recombinant Celf3 and obtained a monoclonal antibody (clone 1E7) and polyclonal antiserum that specifically detected this protein (Fig. 2D). 1E7 recognized multiple bands with sizes of 45, 47, 50 and 52 kDa in the samples prepared from Neuro2A cells, whereas the 52 kDa band was relatively weak in the samples prepared from adult brain (Fig. 2D). None of these bands were present upon knockdown of Celf3, suggesting that the signals were derived from isoforms or degraded products of Celf3 (Fig. 2D). We then carried out cross-link immunoprecipitation (CLIP) analysis with the antiserum (Fig. 3A, B), which showed that Celf3 preferentially interacted with the middle region of Gomafu (Fig. 3A). These interactions were only present upon UV cross-linking, suggesting that Celf3 interacts with Gomafu *in vivo* (Fig. 3A). Celf3 also co-immunoprecipitated with 7SK and Malat1, whereas 18S ribosomal RNA was only weakly associated with Celf3 under these conditions (Fig. 3A).

**Figure 4:**
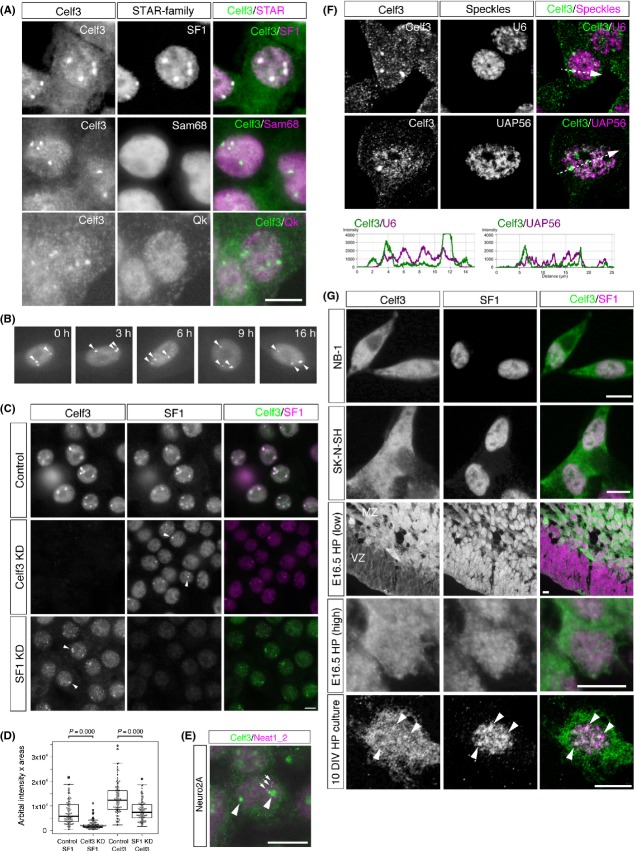
Celf3 and SF1 form CS bodies in Neuro2A cells. (A) Immunofluorescence detection of Celf3 (green) and the STAR family RNA-binding proteins SF1, Sam68 and Qk (magenta) in Neuro2A cells. Note that Celf3 and SF1 are enriched in the same nuclear bodies, whereas Qk and Sam68 are diffusely distributed. (B) Time-lapse imaging of CS bodies visualized by EGFP-Celf3. Note that the number of CS bodies does not change over 16 h. (C) Formation of CS bodies in cells depleted of SF1 or Celf3. Arrowheads indicate the formation of CS bodies in cells lacking either the expression of SF1 or Celf3. (D) Box-and-whisker plot and overlaid beeswarm plot of the size of the CS bodies calculated by multiplying the area of the CS bodies and the average signals of SF1 or Celf3. The circles (*n* = 100) represent the cells measured. (E) Immunofluorescence detection of Celf3 (green) and the paraspeckles marker Neat1_2 (magenta). The CS bodies (arrowheads) did not colocalize with the paraspeckles (arrows). (F) Confocal immunofluorescence detection of Celf3 (green) and the nuclear speckle marker UAP56 and U6 (magenta). The intensity prolife graphs along the line segment shown as dotted lines are given below. (G) Immunofluorescence detection of Celf3 (green) and SF1 (magenta) in NB-1 and SK-N-SH cells and the hippocampal region of an embryonic day 16.5 (E16.5) brain (low: low magnification; high: high magnification) and hippocampal (HP) neurons cultured 10 days *in vitro* (10 DIV). Only subpopulation of arrowheads indicate overlapping signals of Celf3 and SF1. Scale bars, 10 μm.

FISH was carried out to compare the subnuclear distribution of Celf3 and Gomafu in Gomafu-expressing Neuro2A cells and primary culture of hippocampal neurons. As previously reported (Chapple *et al*. 2007; Dev *et al*. 2007), Celf3 was mainly localized both in the cytoplasm and the nucleus (Fig. 3C). Notably, Celf3 in Neuro2A cells accumulated at specific sites in the nucleus and formed nuclear body-like structures (Fig. 3C, arrowheads; see also Fig. 4A). Based on the CLIP results, we initially suggested that Gomafu would accumulate with Celf3. However, peak levels of Celf3 did not coincide with peak levels of Gomafu (Fig. 3C, D). We could not detect prominent co-localization of Celf3 and endogenous Gomafu transcripts in the primary hippocampal neurons that have been cultured for 7 days *in vitro* (Fig. 3C, D). Although we cannot exclude the possibility that the interactions between Celf3 and Gomafu were disrupted during the harsh FISH treatments, these observations suggest that only a small fraction of Celf3 interacts with Gomafu, as has been reported for the other Gomafu-interacting protein SF1 (Tsuiji *et al*. 2011). Accordingly, the amount of Celf3 or SF1 in the biochemical fractions containing Gomafu was below the limit of detection by Western blot (Fig. 3E). Alternatively, the Gomafu-SF1/Celf3 complex could have remained insoluble after the PBS-TX extraction, or the association of these molecules was disrupted even under the mild extraction conditions.

### Celf3 and SF1 are enriched in RNA-dependent nuclear bodies in Neuro2A cells

During the aforementioned studies, we noted that the two Gomafu-associating proteins, Celf3 and SF1, were enriched in the same dots in the Neuro2A cells (Fig. 4A), which we named CS bodies (Celf3 and SF1 nuclear bodies). The CS bodies were clearly detected by immunohistochemistry (Fig. 4A), but the signals were obscured after the harsh FISH treatment (Fig. 3C). The average size of the CS bodies was 0.99 ± 0.28 μm, and 2–3 bodies were typically found in each nucleus (average = 2.2 ± 1.1). Time-lapse imaging of the CS bodies using EGFP-Celf3 showed that the number of nuclear bodies is rather constant in a cell (Fig. 4B, Movie S1 in Supporting Information). We then examined the distribution of Sam68 and quaking (Qk), which belong to the STAR (signal transduction and activation of RNA) family of RNA-binding proteins and are closely related to SF1 (reviewed in Richard 2010). Unlike SF1, Sam68 and Qk were diffusely localized throughout the nucleus and did not localize with Celf3 (Fig. 4A), suggesting that the formation of the CS bodies was specific to SF1 among the STAR protein family.

To investigate whether SF1 and/or Celf3 functioned as structural components of the CS bodies, we used siRNA to individually knockdown these proteins (Fig. 2B, D) and examined the localization of the other protein (Fig. 4C). We observed the formation of CS bodies in the cells lacking the expression of Celf3 or SF1 (arrowheads in Fig. 4C), suggesting that these proteins are not essential architectural components of the CS bodies. However, the sizes of the CS bodies were significantly decreased in the cells depleted of either protein (Fig. 4D), suggesting that Celf3 and SF1 play a role in promoting the formation of CS bodies.

SF1 was previously shown to be localized to the nuclear bodies known as paraspeckles (Choleza 2009). We thus compared the expression of Neat1_2, the core paraspeckle structural noncoding RNA (reviewed in Bond & Fox 2009; Nakagawa & Hirose 2012), with Celf3. The CS bodies did not overlap with Neat1_2 foci (Fig. 4E), suggesting that the CS bodies and paraspeckles were distinct nuclear bodies in the Neuro2A cells. The CS bodies also did not coincide with the distribution of nuclear speckle markers UAP56 and U6 RNA (Fig. 4F).

To investigate whether the CS bodies were ubiquitous nuclear bodies or specific to the Neuro2A cells, we examined the subnuclear localization of Celf3 and SF1 in various cell types. Because Celf3 is mostly expressed in neural tissue (Chapple *et al*. 2007; Dev *et al*. 2007), we used the human neural cell lines NB-1 and SK-N-SH. Celf3 was expressed in a subpopulation of the SK-N-SH cells and ubiquitously expressed in the NB-1 cells, whereas SF1 was ubiquitously expressed in both cell lines. In the NB-1 and SK-N-SH cells that co-expressed Celf3 and SF1, these two proteins were uniformly distributed in the nucleus and did not form CS bodies (Fig. 4G). We then examined the expression of Celf3 in the developing brain. In embryonic day 16.5 (E16.5) hippocampal regions, Celf3 expression was absent in the undifferentiated neural progenitor cells in the ventricular zone and strongly present in postmitotic neurons in the marginal zone (Fig. 4G, E16.5 HP (low)). However, the CS bodies were not present in these cells (Fig. 4G, E16.5 HP (high)). We subsequently examined the expression of Celf3 and SF1 in mature neurons. Because the anti-Celf3 antiserum we obtained was originated from mouse, we used cultured hippocampal neurons to avoid extremely high background staining originating from endogenous IgG when we used adult mouse brain. Although we failed to detect prominent CS bodies, Celf3 and SF1 occasionally accumulated at distinct sites in the nucleus, which was clearly observed under confocal microscopy (arrowheads in Fig. 4G, 10 DIV HP culture). These observations suggest that the CS bodies are not ubiquitous nuclear bodies but rather form in specific cell types under certain conditions.

### CS bodies are RNA-dependent nuclear bodies

**Figure 5:**
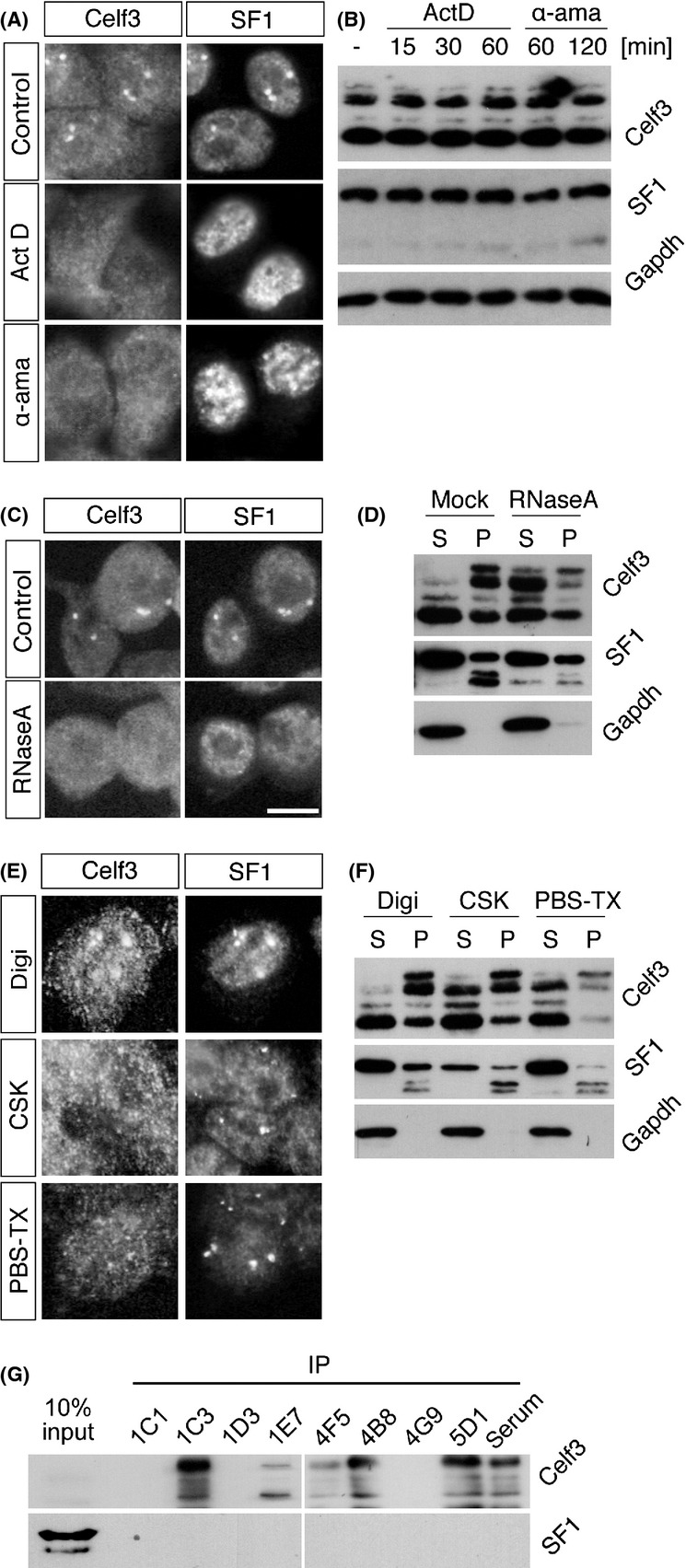
Biochemical characterization of CS bodies. (A) Disruption of CS bodies by transcriptional inhibition. Neuro2A cells were treated with actinomycin D (ActD) and α-amanitin (α-ama) and double-stained for Celf3 and SF1. (B) Western blot analysis of Celf3 and SF1 in cells treated with transcriptional inhibitors. No change was observed in the band patterns of these proteins. (C) CS bodies in RNase-treated cells. Celf3 and SF1 were diffusely distributed throughout the nuclei of RNase-treated cells. (D) Western blot analysis of Celf3 and SF1 in soluble (S) and insoluble (P) fractions prepared from cells treated with RNase A. RNase treatment increased the solubility of Celf3. (E) The sensitivity of the CS bodies to various extraction reagents. The cells were treated with digitonin (Digi), CSK and PBS-TX, and Celf3 and SF1 were simultaneously detected via immunohistochemistry. Celf3 in the CS bodies disappeared after CSK and PBS-TX treatment, whereas SF1 in the bodies was intact. (F) Western blot analysis of Celf3 and SF1 in the soluble (S) and insoluble (P) fractions prepared from cells treated with various extraction reagents. Most of the Celf3 was extracted with PBS-TX, whereas high-mobility SF1 remained insoluble after the extractions. (G) SF1 did not co-immunoprecipitate with Celf3. Eight different monoclonal antibodies that could recognize Celf3 by immunohistochemistry were used to precipitate Celf3. All of these antibodies failed to co-precipitate SF1. Scale bars, 10 μm.

Recent studies have shown that certain nuclear bodies, such as Cajal bodies and paraspeckles, are built on actively transcribed RNAs (Mao *et al*. 2011a; Shevtsov & Dundr 2011). To determine whether this occurs in the CS bodies, we treated the cells with transcriptional inhibitors. Upon transcriptional inhibition, Celf3 was uniformly distributed throughout the nucleus, and SF1 formed discrete speckles (Fig. 5A), whereas the amounts and sizes of the proteins separated by SDS-PAGE were not affected (Fig. 5B). In addition, RNase treatment also disrupted the CS bodies (Fig. 5C), suggesting that ongoing transcription of RNA is required for the formation of CS bodies. To further examine the biochemical properties of the CS bodies, we extracted the cells with multiple nonionic detergents. For these experiments, we did not homogenize the nuclei before extraction, and particular forms of Celf3 and SF1 were insoluble (Fig. 5D, F), unlike the preparation used in the sucrose density gradient analysis (Fig. 3E). Permeabilization of the plasma membrane with digitonin treatment did not largely alter the distribution of Celf3 and SF1, and the CS bodies were clearly observed after the treatment (Fig. 5E). By contrast, Triton X-100 treatment in CSK extracted Celf3 but not SF1 from the CS bodies (Fig. 5E). PBS-TX treatment, which efficiently solubilized the Gomafu complex (Fig. 1A), extracted Celf3 and SF1 from the nucleoplasm, leaving SF1 in the CS bodies (Fig. 5E). These observations suggest that SF1 interacts more strongly with the CS bodies than does Celf3. Interestingly, insoluble SF1 migrated faster than the soluble form according to Western blot analysis (Fig. 5F), suggesting that the CS bodies contained specific isoforms of SF1. Although a certain portion of SF1 remained insoluble in the CS bodies after extraction, we determined whether Celf3 and SF1 interacted in the soluble fraction by performing immunoprecipitation. We used eight monoclonal antibodies and a polyclonal antibody against Celf3; six of these antibodies could immunoprecipitate Celf3. However, SF1 did not co-immunoprecipitate with Celf3 (Fig. 5G), suggesting that Celf3 and SF1 do not interact in the soluble biochemical fraction.

### Localization of Celf3 to CS bodies is regulated by a linker region of Celf3 independent of its RNA recognition motifs

**Figure 6:**
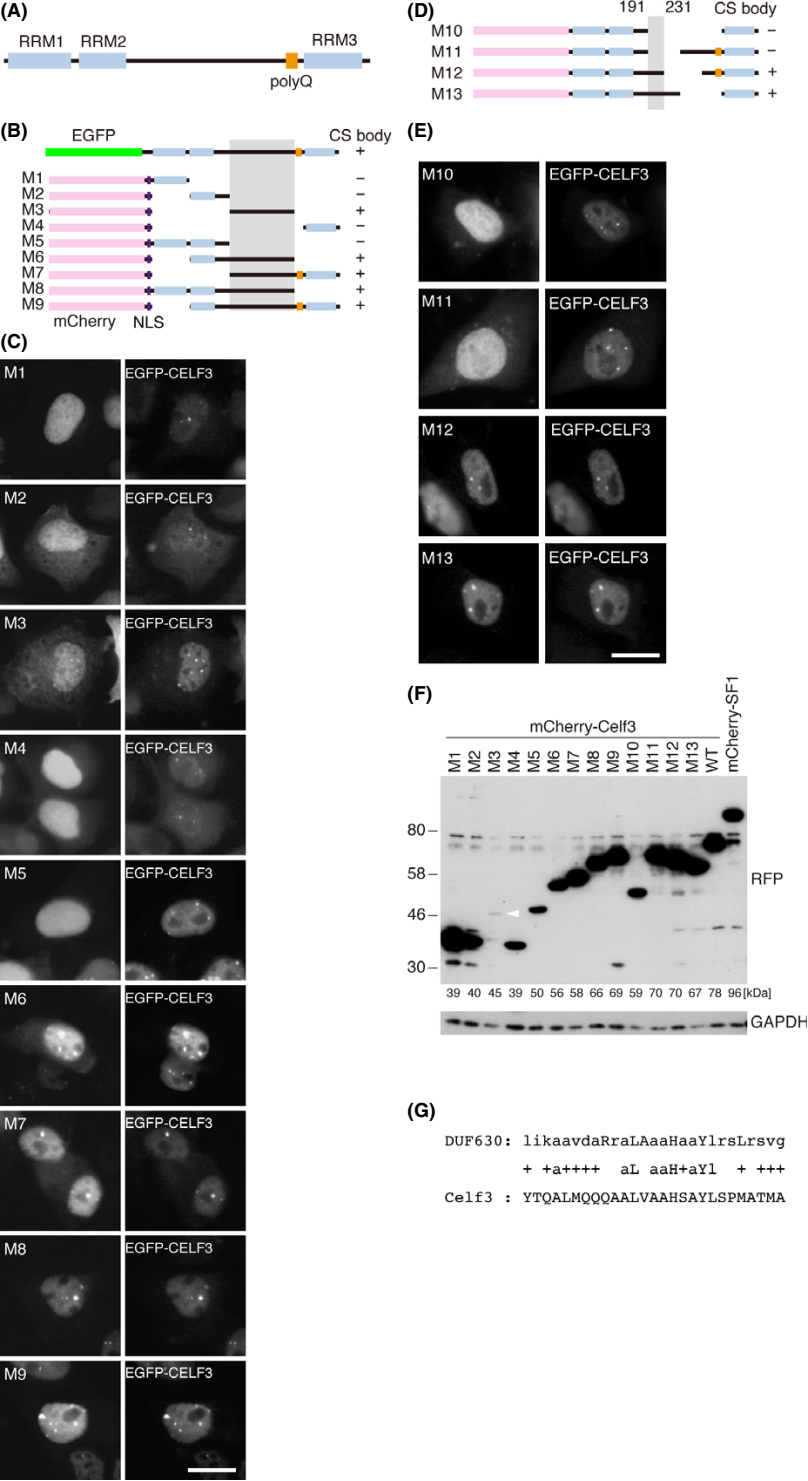
Linker domain of Celf3 regulates the localization of Celf3 in the CS bodies. (A) Schematic representation of the domain structure of Celf3. RRM; RNA recognition motif, polyQ; polyglutamine motif. (B, D) The structures of the deletion constructs of Celf3. Full-length Celf3 was fused to EGFP, and the deletion mutants were fused to mCherry. Shadow represents the motif shared between the molecules localizing to the CS bodies. NLS; nuclear localization signal. (C, E) Simultaneous detection of the mCherry-tagged Celf3 mutants (M1 to M13) and EGFP-tagged full-length Celf3. (F) Western blot analysis of the Celf mutants detected by the anti-mCherry antibody. The calculated molecular mass of each protein is indicated below. The band for M3 (white arrowhead) was weaker than those for the other constructs for unknown reasons. (G) Weak homology of the linker region to DUF630. ‘+’ indicates similar amino acid sequences. Scale bars, 10 μm.

**Figure 7:**
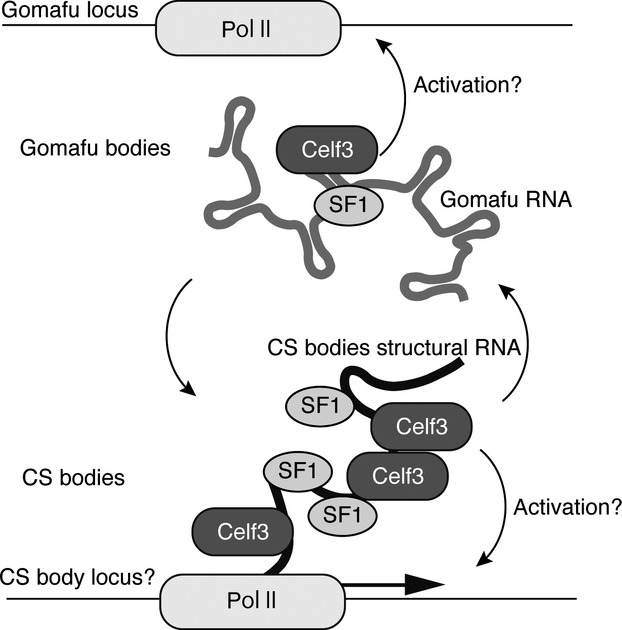
Model of the molecular mechanism of Gomafu. Gomafu forms RNA–protein complexes containing the splicing factors SF1 and Celf3. These splicing factors assemble on transcription sites of specific RNAs to form CS bodies. Gomafu may sequester Celf3 and SF1 and control their dynamics in the nucleus. Celf3 may also affect the expression of other genes such as structural RNA in the CS bodies through the association with their transcripts.

Finally, we identified sequence motifs that regulated the localization of Celf3 to CS bodies using a series of Celf3 deletion mutants (M1–M13) (Fig. 6A, B, D and F). Celf3 contains three RNA recognition motifs (RRMs), and the second and third RRMs are connected with a linker domain that does not contain any conserved protein motifs, with the exception of polyglutamine sequences (Fig. 6A). The mutant molecule containing the linker domain with either of the flanking RRMs (M6–M9) was efficiently enriched in the CS bodies (Fig. 6C; M6–M9). Unexpectedly, a mutant molecule consisting of the linker sequence alone was also enriched in the CS bodies (Fig. 6C; M3), suggesting that the RRM domains are not required for the localization of the CS bodies. Further deletion analysis (Fig. 6D, and F) showed that amino acids 192–231 were common to the mutant molecules that were enriched in the CS bodies (Fig. 6E; M12 and M13). We speculated that certain functional sequence motifs were present in this region. A Pfam (http://pfam.sanger.ac.uk/search) search using these sequences indicated a weak homology to DUF630 (domain of unknown function 630) (Fig. 6G), although this homology was not significant according to the default statistical settings (Fig. 7).

## Discussion

In this paper, we identified Celf3 as a novel Gomafu-interacting protein by screening a custom siRNA library against abundant RNA-binding proteins. While CLIP analysis showed clear binding of Celf3 to the middle regions of Gomafu, FISH analysis indicated that the subnuclear distribution of these two molecules is largely different. We previously identified SF1 as a Gomafu-binding protein and showed that the localization of SF1 is largely different from that of Gomafu (Tsuiji *et al*. 2011). It is intriguing that these two Gomafu-binding proteins, Celf3 and SF1, were assembled in distinct nuclear bodies, which we named CS bodies, in Neuro2A cells. Prominent formation of the CS bodies was observed in this particular mouse cell line but not in two other human-derived neuroblastoma cell lines. However, Celf3 and SF1 occasionally colocalized in cultured hippocampal neurons. We thus speculated that Celf3 and SF1 cooperated to regulate certain nuclear processes in a cell type-specific manner, as observed in the Neuro2A cells. Alternatively, the CS bodies are species-specific nuclear bodies found only in the mouse cells. The CS bodies were sensitive to transcriptional inhibition and RNase treatment. Neuro2A cells might over-express certain gene(s) that contain(s) multiple binding sites for these RNA-binding molecules, leading to the assembly of CS bodies, most likely at the transcriptional site. This hypothesis is consistent with our observation that the number of CS bodies per cell was constant and mostly ranged from two to four, which might reflect the copy number of the gene loci in this cell line. The identification of the architectural RNA components in the CS bodies is a crucial step in elucidating the physiological significance and relevance of the nuclear bodies.

Whereas Celf3 and SF1 were clearly enriched in the CS bodies, the mechanism behind the recruitment of these two proteins to the CS bodies remains unknown. Interestingly, an insoluble form of SF1 that migrates faster than the soluble form on SDS-PAGE was enriched in the CS bodies. It has been reported that the alternative splicing of *SF1* produces several isoforms (Arning *et al*. 1996) and that the phosphorylation of SF1 inhibits or enhances its binding to the splicing factor U2AF depending on the phosphorylation site (Wang *et al*. 1999; Manceau *et al*. 2006). Although we do not know which molecular form of SF1 is localized to the CS bodies, it is possible to obtain functional insight by identifying the SF1 isoform in the nuclear bodies. We have identified the linker sequences of Celf3 as essential elements that regulate its localization to the CS bodies. The linker domain displayed weak homology to DUF630, which is found at the N-terminus of plant bZIP proteins. Notably, DUF630 is suggested to be involved in nucleic acid binding according to a structural modeling study (Rigden 2011). It is thus possible that the linker sequences might cooperate with other RRM domains and provide the specificity for binding the architectural RNA component of the CS bodies. Alternatively, the linker sequences might bind to other molecules that recruit Celf3 to the CS bodies.

Aside from the physiological significance of the formation of CS bodies, the possible functions of Gomafu remain unknown. A series of recent studies reported that nuclear lncRNAs might function as ‘molecular sponges’ that competitively bind to protein factors and thereby negatively regulate their function (Jolly & Lakhotia 2006; Tripathi *et al*. 2010; Yang *et al*. 2011; Nakagawa & Hirose 2012; Yin *et al*. 2012). We thus speculate that Gomafu indirectly regulates Celf3 and SF1, which function in distinct sites in the nucleus, including CS bodies, by forming separate complexes (Fig. 7). A very recent study reported that Gomafu expression is dynamically regulated by neuronal activity; depolarization in cultured neurons leads to the rapid down-regulation of Gomafu within 30 min. This down-regulation is recovered within 2 h (Barry *et al*. 2014). More interestingly, knockdown or over-expression of Gomafu affects schizophrenia-associated alternative splicing of DISK1 or ERBB4 (Barry *et al*. 2014). It should be stressed that the Gomafu-associating proteins SF1 and Celf3 are well-recognized pre-mRNA splicing factors (Kramer 1992; Ladd *et al*. 2001; Suzuki *et al*. 2002; Chapple *et al*. 2007; Ohno *et al*. 2008; Corioni *et al*. 2011). In this context, it is important to determine whether Gomafu regulates the kinetics or dynamics of SF1 and Celf3 within the nucleus and the alternative splicing of target genes regulated by these splicing factors. Nonetheless, we did not observe clear co-localization of Celf3 and Gomafu in any of the cell types we examined. Thus, the interaction of Gomafu and Celf3 might be transient; the two molecules are separately distributed in the nucleus, which is reminiscent of the predominant localization of the splicing factors in the nuclear speckles separated from the transcription sites where the actual splicing reaction occurs (Spector & Lamond 2011).

While knockdown of Celf3 leads to the down-regulation of exogenous Gomafu, the precise mechanism remains unknown. The half-life of the Gomafu transcripts was not affected by Celf3 depletion, suggesting that Celf3 regulates the expression of Gomafu at the transcriptional level. Because we observed the down-regulation of Gomafu in two distinct cell lines that express Gomafu under the control of different promoters (CAG promoter and TRE promoter), it is less likely that Celf3 controls the activity of specific promoter sequences. It should be noted that the sizes of the CS bodies were significantly decreased upon Celf3 knockdown. Thus, Celf3 might also regulate the expression of the architectural RNA of CS bodies. It is intriguing to test whether Celf3 regulates gene transcription by recruiting certain transcriptional activator complexes through the association of nascent transcribed RNAs that are in close proximity to their transcriptional sites.

During the development of the central nervous system, Gomafu is not expressed in undifferentiated progenitor cells but is initially expressed in a subset of postmitotic neurons (Sone *et al*. 2007). This expression pattern of Gomafu correlates with Celf3 expression, although Celf3 is more broadly expressed in almost all of the postmitotic neurons in the brain (Shinichi Nakagawa, unpublished observation). In adults, the expression of Celf3 and Gomafu is mostly restricted to the nervous system at the tissue level (Ladd *et al*. 2001; Chapple *et al*. 2007; Dev *et al*. 2007; Sone *et al*. 2007), supporting the importance of the close correlative expression of these two molecules. It is thus possible that Celf3 expression is a prerequisite for the stable expression of Gomafu during the development and in the adult brain. Celf3 belongs to the Celf family of RNA-binding proteins, which includes six members in mouse. Celf1 and Celf2 are ubiquitously expressed in most of the cells examined, whereas the other members (Celf3 - 6) are neural specific (reviewed in Ladd 2013). Although Celf3 knockout mice do not exhibit obvious neuronal phenotypes (Dev 2006), it would be intriguing to study the expression of Gomafu in the Celf3 knockout mice or in neurons depleted of all of the neuronal Celf family RNA-binding proteins.

The nuclear lncRNAs Xist and Gomafu tightly associate with insoluble materials and are insoluble under nondenaturing buffer conditions. These characteristics have obstructed the biochemical analysis of these lncRNAs (Clemson *et al*. 1996; Sone *et al*. 2007). However, we observed that the use of phosphate buffer greatly increased the solubility of the lncRNAs Gomafu, Xist and Neat1. These complexes can be specifically separated into different fractions by sucrose density gradient ultracentrifugation, suggesting that the complexes maintain their sizes after fractionation. The combination of biochemical fractionation with more specific affinity purification, such as CHART or CHIRP (Simon *et al*. 2011; Chu *et al*. 2011), may enable the identification of all protein components in the lncRNA complex. In addition, we successfully visualized the Gomafu and Malat1 complexes immobilized on PLL-coated glass slides by conventional fluorescence microscopy. This preparation might provide a conventional platform to directly observe the structure of the lncRNA complex using atomic force or cryo-electron microscopy.

## Experimental procedures

### Extraction of the lncRNA complexes and size fractionation on a sucrose density gradient

For PBS-TX extraction, Neuro2A cells were first treated with Digi Buffer (1 × PBS, 1 mm EDTA, 1 mm DTT, 0.01% digitonin) for 5 min at 4 °C to remove the cytoplasmic fractions. The cells were then resuspended in PBS-TX Buffer (1 × PBS, 1 mm EDTA, 1 mm DTT, 0.5% Triton X-100, 0.1 U/μL RNase inhibitor (#SIN-201, TOYOBO, Japan), 1 × Protease Inhibitors (Nacalai #2595-1, Japan), 10 μg/mL heparin) or CSK Buffer (10 mm HEPES pH 7.4, 100 mm NaCl, 1 mm EGTA, 3 mm MgCl2, 0.3 m Sucrose, 0.5% Triton X-100, 1 mm DTT, 0.1 U/μL RNase inhibitor, 1 × Protease inhibitors, 10 μg/mL heparin), incubated on ice for 10–15 min and passed 10 times through a 28G needle attached to a 1-mL syringe. The lysates were separated by centrifugation at 20,630 ***g*** for 5 min at 4 °C, and the supernatants and pellets were retained as the soluble and insoluble fractions, respectively. RNA from each fraction was extracted using TRIzol (Invitrogen). Sucrose density gradient fractionation was carried out with Gradient Station (Biocomp 153-002) according to the manufacturer's instructions. Briefly, 5 mL of 5% and 30% Sucrose Buffer in PBS-TX without Triton X-100 was mixed by time: 1:36/angle: 81.5/speed: 19 to establish a 5–30% sucrose gradient. The PBS-TX extract was loaded onto the gradient and ultracentrifuged at 32 000 rpm for 2 h at 4 °C using a himac CP80WX ultracentrifuge with a P40-ST rotor (Hitachi, Japan). The fractions were collected by speed: 0.3/distance: 5/number: 19. For the lithium lauryl sulfate (LDS; #08923-22, Nacalai, Japan) treatment, 0.5% LDS was added to the extract just before the ultracentrifugation. For *in situ* hybridization, the contents of the fractions were attached onto poly-L-lysine-treated slides (Matsunami #S7441, Japan) for 2 h at 4 °C, briefly washed with PBS, fixed overnight with 4% paraformaldehyde in PBS and processed for *in situ* hybridization.

### Vector construction

All primers used for constructing the vectors are listed in Table S2 in Supporting Information. The full-length cDNAs of Celf3 (NM_172434) and SF1 (NM_00111079) were obtained by RT-PCR using cDNA from mouse brain and Neuro2A cells, respectively. For EGFP-tagged Celf3 (EGFP-Celf3), the cDNA was subcloned into the EcoRI/BamHI sites of the pEGFP-C2 vector (Clontech). For the mCherry-tagged proteins used for Celf3 domain mapping, the PCR-amplified fragments from each Celf3 domain were subcloned into the BamHI/SalI sites of the pmCherry-C1 vector (Clontech). Point mutations resistant to the siRNA treatment were introduced by DpnI-mediated site-directed mutagenesis. For transient expression, full-length Gomafu cDNA was subcloned into the HindIII/SalI sites of the pCI vector (Promega), and the cDNAs of Celf3 and EGFP were subcloned into the pCANw vector with an N-terminal FLAG-HA tag. Transfections were carried out using FuGENE HD Transfection Reagent (Promega) according to the manufacturer's instructions.

### Quantitative PCR and statistical analysis

Total RNA was isolated using TRIzol reagent (Invitrogen). To eliminate DNA, the RNA samples were treated with TURBO DNase (Life Technologies). The RNA (0.5 μg) was reverse-transcribed in 10 μL reactions with ReverTra Ace qPCR RT Master Mix (Toyobo), and 0.5 μL of the products was used in 20 μL quantitative PCR (qPCR) reactions using the Thunderbird SYBR qPCR Mix (Toyobo) on an ABI 7900HT (Applied Biosystems). The PCR conditions were as follows: 95 °C for 1 min, followed by 40 cycles of 95 °C for 15 s and 60–64 °C for 45 s. The threshold cycle (Ct) and baseline were determined automatically using the sds 2.3 software (Applied Biosystems). Standard curves were generated using serial fourfold dilutions of the control samples. The standard curve had an efficiency of 90% to 110%, which corresponds to a slope of 3.58 to 3.10 and a coefficient of correlation (R2 value) greater than 0.99. The specificity of each primer pair was verified using dissociation curve analysis. Xist RNA was used for normalization. The primer pairs were designed using Primer3 Plus (http://www.bioinformatics.nl/cgi-bin/primer3plus/primer3plus.cgi).

### *In situ* hybridization and immunohistochemistry

Fluorescent *in situ* hybridization (FISH) was carried out as previously described (Sone *et al*. 2007). Briefly, the samples were fixed on culture slides, treated with 0.1 N HCl and Proteinase K, fixed and acetylated. After prehybridization at 55 °C for 0.5–2 h, the slides were hybridized with digoxigenin (DIG)- and fluorescein-labeled RNA probes at 55 °C overnight. The hybridized slides were washed at 55 °C, treated with RNase A and intensively washed with low ionic buffer at 55 °C. The DIG and fluorescein probes were detected with mouse anti-DIG/anti-mouse IgG Cy3 antibodies and rabbit antifluorescein/anti-rabbit IgG Alexa Fluor 488 antibodies, respectively. The following probes were used: Gomafu middle fragment (Tsuiji *et al*. 2011), Malat1 AK141413 (Nakagawa *et al*. 2012), Xist AK039861 (Sone *et al*. 2007), Neat1_1 AV089414 (Nakagawa *et al*. 2011), NEAT1_2 (Nakagawa *et al*. 2011) and Gapdh AK144690. For immunofluorescent (IF) detection of proteins, Neuro2A cells grown on the slides were fixed for 30 min at 4 °C with 4% paraformaldehyde (PFA)/Hank's balanced salt solution (HBSS), permeabilized in 100% methanol for 5 min at −20 °C and incubated with 4% skim milk (Difco) in 1 × PBS containing 0.1% Tween-20 (PBST). The slides were sequentially incubated with primary antibodies and fluorescent conjugated secondary antibodies. For transcriptional inhibition, 0.5 μg/mL actinomycin D (Sigma #A9415) and 10 μg/mL α-amanitin (Sigma #A2263) were added to the culture medium and incubated at 37 °C for 0.5 h and 2 h, respectively. For RNase A treatment, Neuro2A cells were grown on plastic dishes, incubated in RNase A sol (100 μg/mL RNase A, 0.01% digitonin in CSK Buffer without Triton X-100) on ice for 5 min, washed and fixed. Fluorescent images were obtained using an epifluorescent microscope (BX51, Olympus) equipped with a CCD camera (DP70) or by performing confocal microscopy (Zeiss, LSM-Pascal). For quantitative analysis of the Celf3 and SF1 signals, the images were analyzed using Image J. For time-lapse imaging of CS bodies, Neuro2A cells were transiently transfected with EGFP-Celf3, and time-lapse images were obtained using LCV110 (Olympus, Japan) at the time interval of 10 min.

### Immunoprecipitation and Western blotting

Immunoprecipitation (IP) was carried out with Dynabeads Protein G (Life Technologies) according to the manufacturer's instructions with some modifications. Briefly, the antibody was immobilized on the Dynabeads, incubated with PBS-TX, washed and boiled in SDS sample buffer to elute the bound proteins. For the efficient detection of Celf3 by Western blot analysis (WB), the monoclonal antibody 1E7 was cross-linked to the Dynabeads with dimethyl pimelimidate (DMP) or bis (sulfosuccinimidyl) suberate (BS3) to reduce the elution of the IgG heavy chains, which interferes with the Celf3 signal. Crosslink-IP (CLIP) was carried out as previously described (Hasegawa *et al*. 2010) with some modifications. Briefly, the Neuro2A cells were UV-irradiated at 400 mJ/cm^2^ in ice water, lysed in buffer containing 1% LDS, 0.1 mg/mL BSA, 0.1 mg/mL yeast tRNA and 10 μg/mL heparin and sonicated with a Bioruptor (Cosmo Bio #UCD-250, Japan) for five cycles of 30 s at 250 W at 30-s intervals. The lysate was then diluted fivefold to adjust the LDS concentration to 0.2%, centrifuged to remove insoluble precipitates, precleared with Protein G Agarose (Millipore) and incubated with an antibody immobilized on Dynabeads. After extensive washes, the RNA contents in the immunoprecipitates were extracted by Proteinase K digestion, treated with TurboDNase and reverse-transcribed for qPCR analysis. For the RNA purified from the input extract, additional purification using the RNeasy MinElute kit (QIAGEN) was carried out. Immunodetection of the proteins by Western blot analysis (WB) was carried out according to a standard procedure. The specific detection of RNA by Northern blot analysis (NB) was carried out using DIG/fluorescein labeling and detection systems (Roche) according to the manufacturer's instructions.

### Production of recombinant proteins and anti-Celf3 monoclonal antibodies

To produce the His-tagged recombinant proteins His-Celf3, the corresponding cDNA was subcloned into the BamHI/SalI sites of the pET-28a vector (Millipore). The recombinant protein was produced in *E. coli* BL21 (DE3) and purified with TALON resin (Clontech). For immunization, His-Celf3 (10–50 μg) was mixed with the adjuvant TiterMax Gold (TiterMax) to produce antigen-adjuvant emulsions (100 μL/mouse) and injected intraperitoneally into four BALB/c female mice (8 weeks old at the first injection) every 2 weeks. The lymphocytes from the immunized mice were fused with myeloma P3U1 cells in a ratio of 3:1 to 5:1 by mixing in 50% polyethylene glycol (Roche). The fused cells were dispersed into 80 mL GIT medium (Wako, Japan) supplemented with 1 ng/mL IL-6 (PeproTech) and 1 × HAT (Kohjin-Bio). The cells were seeded into four 96-well plates at 0.2 mL/well and grown for 10 days at 37 °C. The first screening was carried out by enzyme-linked immunosorbent assay (ELISA) with 50 ng/well of His-Celf3 (37 positive clones were obtained), and the clones were subsequently screened with Western blot, immunofluorescence and immunoprecipitation analyses. As a result, clone 1E7 was selected as the anti-Celf3 monoclonal antibody for use in this study. Antiserum against Celf3 was prepared at the fusion step.

### Antibodies

The primary antibodies used to detect specific proteins were as follows: mouse anti-SF1 antibody (2E12, Abnova H00007536-M01A) for CLIP, IP, IF and WB; rabbit anti-SF1 (Aviva ARP41214_T100) for IF and WB; anti-GAPDH (Millipore #MAB374), antitubulin (Abcam # ab7291), and anti-RFP (MBL #PM005, Japan) for WB; anti-GFP (Roche #1814460) for IP and WB; anti-FLAG M2 (Sigma #F1804) as a mock control for CLIP and IP; mouse anti-DIG (21H8, Abcam #ab420) and rabbit antifluorescein (Abcam #ab19491) for FISH. The secondary antibodies used were as follows: anti-mouse IgG HRP (GE Healthcare #NA931) for ELISA and WB; anti-rabbit IgG HRP (GE Healthcare #NA934) for WB; anti-mouse IgG Cy3 (Millipore #AP124C) and anti-rabbit IgG Alexa Fluor 488 (Life Technologies #A11008) for FISH and IF; and anti-DIG AP (Roche #1093274) and antifluorescein AP (Roche #1426338) for NB.

### Knockdown with siRNA

Knockdown of specific gene expression by siRNA was carried out using Lipofectamine RNAiMAX (Life Technologies) according to the manufacturer's instructions. All siRNAs were purchased from Ambion. The siRNAs used were siCelf3 (siRNA ID# 176186) and siSF1 (siRNA ID# 187640). The sequences of the siRNAs are provided in Table S2 in Supporting Information. Rescue of Gomafu expression in cells treated with siCelf3 was carried out by introducing siRNA-resistant silent mutations in Celf3 (Celf3mut). The expression vectors for EGFP, Celf3 and Celf3mut were co-transfected with a Gomafu expression vector 36 h after the treatment with siCelf3 and incubated for an additional 36 h.

### Measurement of the stability of Gomafu transcripts

For the conditional expression of Gomafu, mEGFP of pT2K-TRE-mEGFP (Tanabe *et al*. 2006) was replaced with full-length Gomafu to yield pT2K-TRE-Gomafu. Initially, Neuro2A cells that stably express Tet-OFF (Clontech) were established. The cells were subsequently co-transfected with pT2K-TRE-Gomafu and pCAGGS-T2TP (Tanabe *et al*. 2006), and clones of Neuro2A cells that stably expressed Gomafu under the control of the tetracycline-responsive element were selected. Doxycycline was added at the concentration of 0.5 μg/mL to shut off the promoter activity, and total RNAs were recovered at different time points using TRIzol (Invitrogen). The expression of Gomafu was measured by qPCR using three biological triplicates. *Actb* was used for normalization.

## Supporting Information

Movie S1 Time-lapse imaging of CS bodies.

Table S1 Expression of Gomafu in Neuro2A cells upon knockdown of various RNA binding proteins. Expression was norlized with 7SK

Table S2 Primers and siRNAs used in the present study
